# Stability performance analysis of Fe based MOFs for peroxydisulfates activation to effectively degrade ciprofloxacin

**DOI:** 10.3389/fbioe.2023.1205911

**Published:** 2023-07-28

**Authors:** Fang-Lan Geng, Hai-Yuan Chi, Hua-Chao Zhao, Jin-Quan Wan, Jian Sun

**Affiliations:** ^1^ State Key Laboratory of Environmental Chemistry and Ecotoxicology, Research Center for Eco-Environmental Sciences, Chinese Academy of Sciences, Beijing, China; ^2^ Institute of Environmental Research at Greater Bay Area, Key Laboratory for Water Quality and Conservation of the Pearl River Delta, Ministry of Education, Guangzhou University, Guangzhou, China; ^3^ College of Environment and Energy, South China University of Technology, Guangzhou, China

**Keywords:** Fe-based metal-organic frameworks, peroxodisulfate, organic micropollutants, stability performance, crystal structure

## Abstract

Fe-based metal-organic frameworks (MOFs) show high activity toward the activation of peroxodisulfate (PDS) for the removal of organic micropollutants (OMPs) in wastewater treatment. However, there is a phenomenon of Fe ion dissolution in the Fe-based MOFs’ active PDS system, and the reasons and influencing factors that cause Fe ion dissolution are poorly understood. In this study, we synthesized four types of Fe-based MOFs and confirmed their crystal structure through characterization. All types of Fe-based MOFs were found to activate PDS and form sulfate radicals (SO_4_^−^), which effectively remove OMPs in wastewater. During the process of Fe-based MOFs activating PDS for CIP removal, activated species, oxidant reagent, and pH negatively impact the stability performance of the MOFs’ structure. The coordination bond between Fe atom and O atom can be attacked by water molecules, free radicals, and H^+^, causing damage to the crystal structure of MOFs. Additionally, Fe (II)-MOFs exhibit the best stability performance, due to the enhanced bond energy of the coordination bond in MOFs by the F ligands. This study summarizes the influencing factors of Fe-based MOFs’ damage during PDS activation processes, providing new insights for the future development of Fe-based MOFs.

## Introduction

Metal-organic frameworks (MOFs), as new nano-materials, have a periodic crystal structure composed of inorganic metal ions at the center and organic bridging ligands connected through coordination bonds (Kuppler et al., 2009). The hydrothermal method uniformly disperses metal central ions and organic ligands in a solvent to synthesize MOF crystals through a high-temperature and high-pressure reaction process (Han et al., 2012). Compared with traditional inorganic materials, MOFs have the following advantages (Lamia et al., 2009; Zhu et al., 2019a): Excellent pore structure and ultra-high specific surface area make them have great potential for adsorption. The organic ligands uniformly dispersed on the surface can facilitate the introduction of other organic functional groups to modify the structure of MOFs, giving them different functional characteristics. The incomplete coordination of MOFs will form Lewis acid active sites, namely, unsaturated active sites (CUS), which make MOFs have good application prospects as catalysts. Among them, the CUS in MOFs has the characteristics of diversity, repeatability, and efficiency, attracting more attention in the field of catalysis (Lamia et al., 2009; Sun et al., 2016). Currently, the most widely studied MOFs in the field of catalysis are the MILs series. The MIL series are synthesized using Fe or Al ions and organic ligands like terephthalic acid to form a polyhedral crystal structure, which has many active sites of Lewis acid and shows excellent potential (Gao et al., 2017). Among them, the MOFs composed of Fe metal central ions exhibit the best performance because they are composed of non-toxic and harmless Fe ions, making them environmentally friendly (Yu et al., 2019). Additionally, Fe metal central ions can form various coordination modes such as four coordination or six coordination, which can easily form CUS in the absence of organic ligands (Sun et al., 2022). Therefore, Fe-based MOFs have been widely attempted in the field of catalysis.

Fe-based MOFs have been widely used in photocatalysis, electrocatalysis, and advanced oxidation processes (AOPs). In recent years, AOPs based on persulfate systems have shown significant advantages in degrading organic micropollutants (OMPs) (Al Hakim et al., 2019; Zhang et al., 2020a; Duttagupta et al., 2020; He et al., 2021). These advantages include high oxidation-reduction potential; simultaneous degradation of multiple OMPs; simple operation at room temperature and pressure, and being environmentally friendly, making them widely used in degrading OMPs in natural water bodies. Considering these advantages, considerable efforts have been dedicated to applying Fe-based MOFs to activate persulfates for OMP removal. For example, Sun et al. applied Fe-MIL-53 to activate peroxodisulfate (PDS) for OMPs removal in wastewater and found that changes in synthesis temperature and time could affect the content of CUS in MOFs (Sun et al., 2022). Andrew Lin et al. (2015) used Fe-MIL-88A to successfully degrade rhodamine B through activating PDS and proposed that the hexagonal rod structure increases the exposure area of its activation site. Xu et al. (2020) combined Fe-MIL-101 and reduced graphene, utilizing the reducing ability of reduced graphene to accelerate the activation performance of MOFs. Chi et al. (2019) synthesized rod-like Fe (II)-MOFs with ferrous ions and showed that MOFs synthesized by ferrous ions can indeed degrade small molecule organic substances in a relatively short time. Additionally, Chi et al. (2021a) constructed a molecular imprinting layer on the surface of Fe (II)-MOFs, which can enrich and specifically recognize the target contaminant to enhance the removal ability. However, previous reports have mentioned that there is a certain amount of Fe ion dissolution during Fe-based MOFs activated PDS systems. The reasons and influencing factors causing iron ion dissolution have not been clearly identified. Previous studies have shown that water molecules can attack the connection bonds between the metal central ions and organic ligands of Fe-based MOFs in humid environments, causing the structure of MOFs to crack and leading to the dissolution of metal central ions (Burtch et al., 2014; Liu et al., 2014; Liu et al., 2015). In advanced oxidation processes, Fe-based MOFs are exposed to the water environment for a long time, so their water stability is an important factor affecting their recycling performance. Additionally, various active species with strong oxidative ability are present, and it is unknown whether they will have a destructive effect on the crystal structure of Fe-based MOFs (Chi et al., 2020). Therefore, it is necessary to summarize the influence of the reaction conditions on the stability performance, which can lay the foundation for further modifications of the structure of Fe-based MOFs.

In this study, four types of Fe-based MOFs were synthesized using the hydrothermal method. The structural characteristics of these MOFs were summarized through characterization analysis. The differences in their activation performance were analyzed by activating PDS to degrade ciprofloxacin (CIP). (CIP, an antibacterial drug widely used in areas such as fungicides and disinfectants, poses a significant threat to biological systems (Liu et al., 2018; Hu et al., 2020). These antibiotics have a devastating impact on the survival of most microorganisms, reducing the efficiency of anaerobic technology, enzyme engineering, and other biotechnologies (He et al., 2021). Therefore, it is crucial to find an effective method to mitigate the biothreat caused by CIP. Furthermore, the effect of different catalytic conditions on the structure of MOFs was evaluated using dissolved iron concentration as the standard. Finally, the damage mechanism of Fe-based MOFs under advanced oxidation processes was summarized by comparing the characteristic peaks of functional groups in Fourier transform infrared spectra (FT-IR) before and after the reaction. This research provides new insights into the development of multifunctional and stable Fe-based MOFs.

## Materials and methods

### Chemicals

The chemicals used in this study were purchased from the chemical company without further purification. Ciprofloxacin (CIP, 99%), p-phthalic acid (1,4-BDC, 99.0%) N, N-dimethylformamide (DMF, HCO(CH_3_)_2_, 99.5%), dimethyl sulfoxide (DMSO) and fumaric acid were obtained from the Aladdin Chemistry Co., Ltd. (Shanghai, China). Ferrous chloride (FeCl_2_·4H_2_O, 99.0%), Ferric chloride hexahydrate (FeCl_3_·6H_2_O, 99.0%), persulfate (PDS, Na_2_S_2_O_8_, 98.0%), and Hydrofluoric acid (HF, 4%) were supplied by from Sinopharm Chemical Reagent Co., Ltd. (Beijing, China). Methyl alcohol (MeOH, CH_4_O, 99.5%) and ethanol (C_2_H_6_O, 99.7%) were supplied by Guangzhou Chemical Reagent Factory (Guangzhou, China). The water used in the test was purified by a Millipore reverse osmosis (RO) system.

### Synthesis of the Fe-based MOFs

Fe (II)-MOFs were synthesized following our previous work (Chi et al., 2019). Specifically, 13.50 mmol FeCl_2_·4H_2_O, 5.40 mmol 1,4-benzenedicarboxylic acid (H_2_BDC), 250 mL dimethylformamide (DMF), and 30 mL methanol were placed in a 500 mL three-neck flask. Following this, 8 mL HF (concentration: 40%) was slowly added to the mixture solution. The solution was purged with dry nitrogen for 10 min, stirring at 140°C for 24 h under a nitrogen atmosphere. The resulting precipitate was purified with methanol and dried in a vacuum furnace at 50°C for 12 h to obtain Fe (II)-MOFs.

Fe-MIL-53 was synthesized according to the method reported by Sun et al. (2022). In brief, 5 mmol FeCl_3_·6H_2_O and 5 mmol 1,4-BDC were evenly dissolved in 25 mL DMF. The mixed solution was poured into a 100 mL Teflon-lined steel autoclave, and heated at 150°C for 5 h. The sample was washed with methanol and finally dried under vacuum at 50°C for 12 h. The synthesized material was designated as Fe-MIL-53.

The synthesis process of Fe-MIL-88A was conducted according to the method reported by Andrew Lin et al. (2015). First, 8.4 mmol fumaric acid and 8.4 mmol FeCl_3_·6H_2_O were dissolved in 42 mL ultrapure water, using ultrasonic treatment. The resulting mixed solution was then transferred to a 100 mL Teflon-lined steel autoclave and heated at 85°C for 2 h. The resulting solid powder was washed with ethanol and ultrapure water and then dried for 12 h under vacuum conditions at 100°C. The synthesized material was designated as Fe-MIL-88A.

The synthesis of Fe-MIL-101 was prepared using a solvothermal method according to the procedure described by Skobelev et al. (2013) In this method, 2.5 mmol FeCl3·6H2O and 1.25 mmol 1,4-BDC are dissolved in 25 mL DMF. The resulting mixture was then transferred to a 100 mL Teflon-lined steel autoclave and placed in a fan oven preheated to 110°C, where it was maintained for 24 h. The product was subsequently washed three times with ethanol and dried in a vacuum oven at 150°C for 12 h to obtain Fe-MIL-101.

### Characterization of Fe-based MOFs

The crystallite structures were analyzed using an X-ray diffractometer, specifically the D8 Advance X-ray Diffraction system (XRD), with a scan speed of 2°/min and a step size of 0.02° in 2θ. The morphology was observed using surface morphological properties, and elemental distribution was examined using a ZEISS Merlin field emission scanning electron microscope (FESEM) equipped with energy-dispersive X-ray spectroscopy (EDS). The surface functional group structure was determined via Fourier transform infrared spectra (FT-IR) using a Nicolet Magna 550 FT-IR spectrometer.

### Evaluation of catalytic performance

In a typical degradation experiment, a specific number of Fe-based MOFs was added to a 250 mL conical flask containing a mixture solution of CIP and PDS. At various time intervals (0, 10, 20, 30, 40, 60, 90, and 120 min), 1.0 mL of the solution was collected from the flask and immediately mixed with an equal amount of pure ethanol to quench any radical reactions for subsequent analysis. All samples were then filtered using a 0.22 μm water phase filter to separate the supernatant liquid from the catalysts for further analysis. The concentration of CIP in the samples was analyzed using high-performance liquid chromatography (HPLC) (Shimadzu LC-20A, Japan). An Agilent HPLC system equipped with a reverse-phase Hypersil C-18 column (250 mm × 4.6 mm, i.d., 5 μm particle size) was utilized. The CIP determination was performed at a maximum absorption wavelength of 278 nm. The mobile phase used was a mixture of methanol and water (0.5% acetic acid) in the ratio of 32:68 (v/v) with a flow rate of 1 mL/min and an injection volume of 20 μL at 28°C. The limit of detection (LOD) for CIP was found to be 25.48 μg mL^−1^ and the limit of quantification (LOQ) was determined to be 76.44 μg mL^−1^.

### Determination of Fe ions concentration in the degradation reaction system

The degradation experiment of CIP was performed in a constant temperature shock incubator at 25°C with a speed of 180 rpm. CIP and PDS were dissolved in deionized water at a molar ratio of 1:150. Then, 0.4 g·L^−1^ Fe-based MOFs were added and the suspension was mechanically stirred for 120 min. At the designated time intervals (0, 10, 20, 30, 40, 60, 90, and 120 min), 1.0 mL of the reaction solution was taken and immediately mixed with 1.0 mL of ethanol to quench the free radicals. All samples were filtered using a 0.22 μm water phase filter to analyze the iron concentration. Typically, O-phenanthroline spectrophotometry was used to measure the iron concentration. Specifically, 0.5 mL of the reaction solution was placed in a 25 mL colorimetric tube. Then, 1.0 mL (1 + 3) hydrochloric acid, 5.0 mL of ammonium acetate-glacial acetic acid buffer solution, and 2.0 mL of 0.5% phenanthroline were added. The solution was diluted with distilled water to the scale, shaken well, and left to stand for 15 min. The absorbance (ABS) was determined at 510 nm, and the absorbance was finally converted to the concentration of Fe ions.

### Analysis of electron paramagnetic resonance (EPR)

The reactive radical species generated during the advanced oxidation process were investigated using electron paramagnetic resonance (EPR) analysis. 5,5-dimethyl-1-pyrroline N-oxide (DMPO) was used as an effective spin trapping agent to capture **·**OH, ^**·**^O_2_^−^ or SO_4_^−**·**^, while 2,2,6,6-tetramethylpiperidine (TEMP) was used to effectively detect ^1^O_2_ in EPR experiments. The typical detection process is as follows: 5 mL of ultrapure water, 20 mg Fe-based MOFs, 20 mmol PDS/PMS, and 100 mM DMPO were added to a flat 15 mL container. EPR testing was conducted at 0, 5, and 10 min.

### Radical scavenger experiments

To confirm the function of each active substance in the degradation of CIP, scavenging experiments were performed. Tertiary butanol (TBA) can effectively quench ·OH, with a quenching constant of k = (3.8–7.6) × 10^8^. Ethanol (EtOH) has good quenching effects on ·OH and SO_4_^**−˙**^, with quenching constants of k = (1.2–2.8) × 10^9^ and k = (1.6–7.7) × 10^7^, respectively (Zhu et al., 2019b; Fan et al., 2019). In a typical scavenger experiment, CIP and PDS were dissolved in deionized water at a certain molar ratio. Then, Fe-based MOFs and a specific amount of scavenger were added. At a given time, 1.0 mL of the solution would be taken and immediately filtered using a 0.22 μm water phase filter for the analysis of the CIP concentration.

## Results and discussion

### Characterization analysis of Fe-based MOFs

**FIGURE 1:**
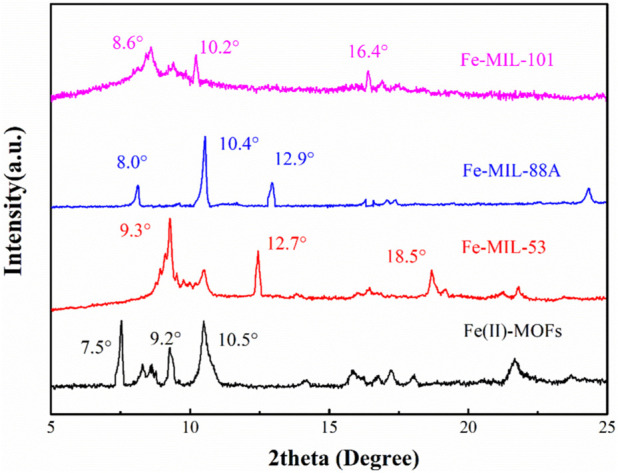
The XRD spectrum of the different Fe based MOFs.

The XRD spectra in Figure 1 depict the different crystal structures of Fe-based MOFs. In the XRD spectra of Fe(II)-MOFs, three distinct characteristic peaks are observed at 7.5°, 9.2°, and 10.5°, which aligns with the description in previously reported literature (Chi et al., 2021a). The XRD spectra of Fe-MIL-53 exhibit three main characteristic peaks at 9.3°, 12.7°, and 18.5°, similar to the Fe-MIL-53 reported by Sun et al. (Sun et al., 2022). The characteristic peaks of MIL-88A are present at 8.0°, 10.4°, and 12.9°, consistent with the Fe MIL-88A reported by Andrew Lin et al. (2015). Similarly, the synthesized Fe-MIL-101 demonstrates a good crystal structure match with the Fe-MIL-101 reported by Duan et al. (2017), with characteristic peaks appearing at 8.6°, 10.2°, and 16.4°, respectively. Additionally, there are various other miscellaneous peaks in the XRD spectra, and the intensity and positions of these peaks differ from those reported in the literature. These variations can be attributed to slight changes in the synthesis conditions during the production of Fe-based MOFs. However, these changes in the miscellaneous peaks have little impact on the fundamental crystal structure of the Fe-based MOFs. Therefore, based on the XRD spectra, it can be concluded that four different Fe-based MOFs were successfully synthesized in this experiment.

**FIGURE 2:**
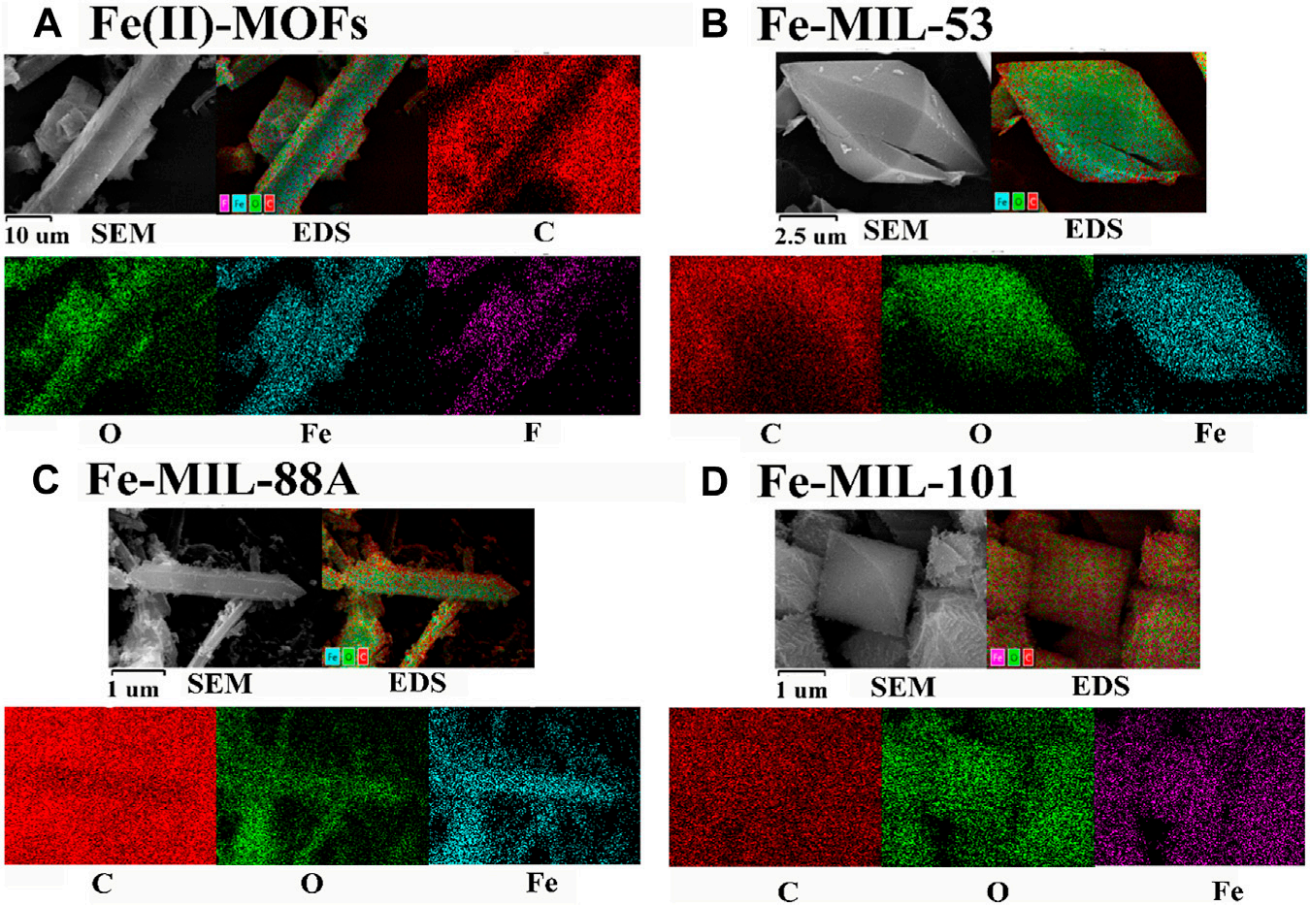
The SEM with EDS-mapping images of the different Fe based MOFs. **(A)** Fe(II)-MOFs. **(B)** Fe-MIL-53. **(C)** Fe-MIL-88A. **(D)** Fe-MIL-101.

The SEM and EDS spectra of Fe-based MOFs are shown in Figure 2. In Figure 2A, Fe(II)-MOFs exhibit a typical rod-shaped structure with lengths ranging from 10 to 100 μm. The surface of the rods shows a uniform distribution of four elements: C, O, Fe, and F. Fe-MIL-53, as depicted in Figure 2B, displays a characteristic spindle structure with dimensions ranging from 10 to 20 μm. On its surface, the elements C, O, and Fe are uniformly distributed. Figure 2C illustrates that Fe-MIL-88A takes the form of a typical dodecagonal prism with crystal sizes between 1 and 10 μm. It is composed of three elements: C, O, and Fe. Similarly, Fe-MIL-101, shown in Figure 2D, consists of three elements: C, O, and Fe. It has a standard octahedral structure with crystal sizes ranging from 0.5 to 2 μm. The surface morphology of the different types of Fe-based MOFs is generally consistent with what has been reported in the literature, with minor variations in crystal size. These differences can be attributed to variations in heating or cooling rates during the synthesis process, which can influence the crystallization process (Bosch et al., 2017). Therefore, the SEM and EDS analyses further support the synthesis of four distinct Fe-based MOF materials with different crystal structures.

### Activation performance of Fe-based MOFs

**FIGURE 3:**
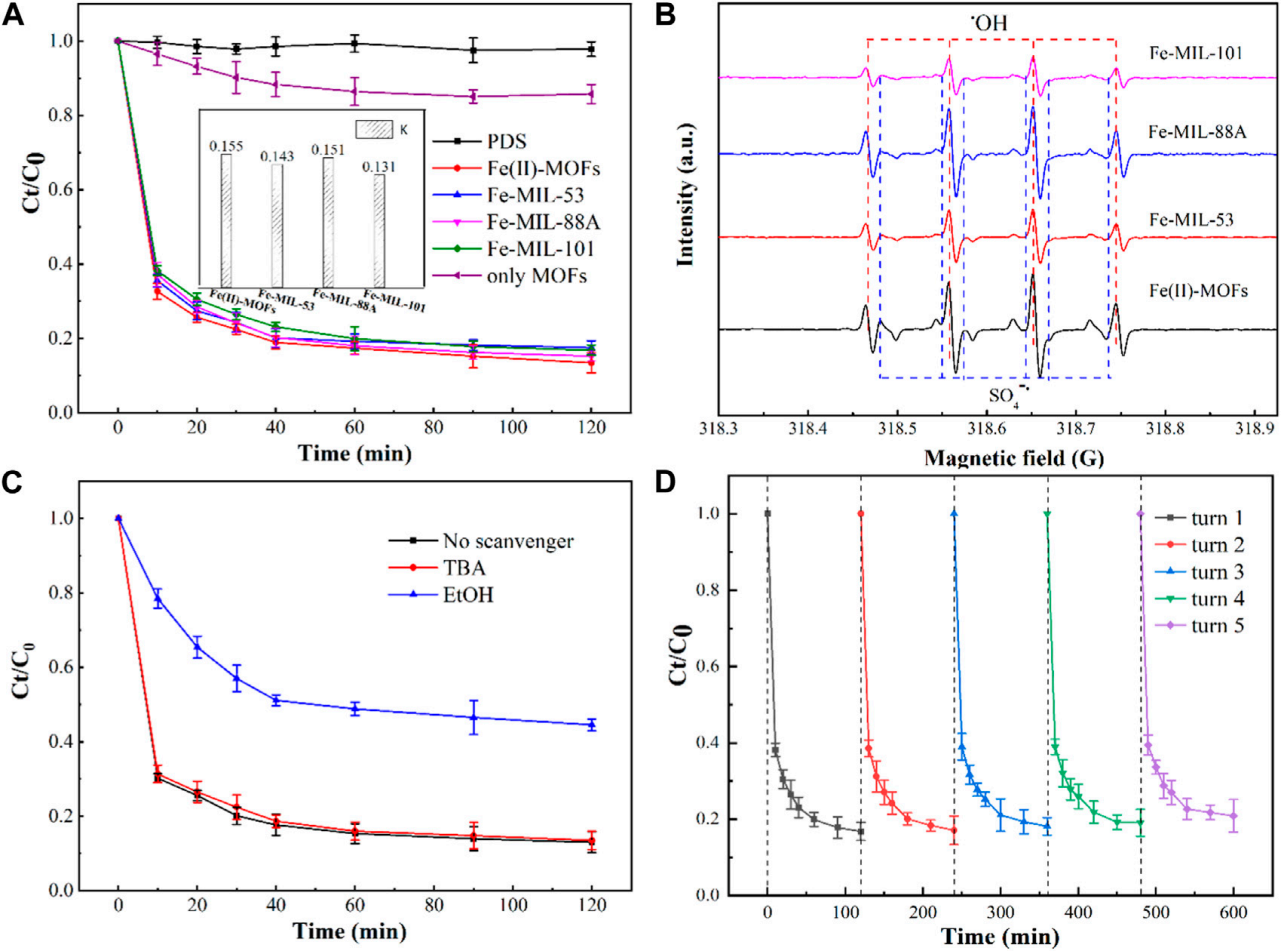
**(A)** Effect of the different iron-based MOFs on CIP degradation performance. Experimental conditions: [CIP] = 0.036 mmol L^-1^, CIP: PDS = 1: 150, catalysts amount = 0.4 g L^-1^, T = 25°C, ambient pH, only MOFs: Fe(II)-MOFs. **(B)** The EPR spectra in different iron-based MOFs/PDS systems. **(C)** Influence of radical scavenger on CIP degradation in different iron-based MOFs/PDS systems. Experimental conditions: [CIP] = 0.036 mmol L^-1^, CIP: PDS = 1: 150, catalysts amount = 0.4 g L^-1^, [TBA]/[EtOH] = 0.1 mol L^-1^, T = 25°C, ambient pH. **(D)** The recycling runs of the degradation of DBP over the Fe (Ⅱ)-MOFs/PDS system. Experimental conditions: [CIP] = 0.036 mmol L^-1^, CIP: PDS = 1: 150, catalysts amount = 0.4 g L^-1^, T = 25°C, ambient pH.

The ability of Fe-based MOFs with different structures to activate PDS for CIP degradation varies, and it is closely related to their morphology and the presence of activation sites. Figure 3A illustrates the ability of Fe(II)-MOFs, Fe-MIL-53, Fe-MIL-88A, and Fe-MIL-101 to activate PDS for CIP degradation. In the absence of a catalyst, PDS alone exhibited limited capability to remove CIP, indicating that the production of active species for CIP degradation from PDS was minimal at room temperature. DBP, on the other hand, was hardly removed in the system containing only MOFs/CIP, indicating that MOFs had poor adsorption properties for CIP. However, we observed that all four different Fe-based MOFs could effectively activate PDS to degrade CIP. This is because the coordinatively unsaturated metal sites (CUS) in MOFs can transfer electrons in PDS to generate active species, which effectively degrade CIP (Chi et al., 2019). The removal rate of CIP was calculated using the pseudo-first-order kinetic equation. The pseudo-first-order kinetic equation is as follows (Chi et al., 2020):

$${\ln(A}_{2}\frac{C_{t}}{C_{0}}+A_{2})=-kt$$ (1)

C_t_ (mg L^−1^): The concentration of CIP when the degradation time is t (min); C_0_ (mg L^−1^): the concentration of CIP at the initial stage; k (min^−1^): Degradation rate constant; A_1,_ A_2_: System error correction constant;

In the pseudo-first-order kinetic model, all the *R* ^2^ values are greater than 0.97, indicating accurate and reliable fitting results. The calculation results reveal that the degradation rate constant of Fe(II)-MOFs is the highest, indicating that Fe(II)-MOFs exhibited the most efficient activation of PDS. This can be attributed to the main active site of Fe(II)-MOFs beings Fe(II)CUS, while the other Fe-based MOFs primarily contained Fe(III)CUS as the main active site. According to literature Fe(II)CUS has higher activation performance compared to Fe(III)CUS, thus explaining the superior activation performance of Fe(II)-MOFs. In the Fe-MIL-53, Fe-MIL-88A, and Fe-MIL-101 systems, their activation performances also differed due to the varying number of exposed Fe(III)CUS exposed on the crystal surfaces (Tang and Wang, 2018). Research has shown a positive correlation between the number of CUS in MOF crystals and the size of the crystal plane (Liao et al., 2019). Analyzing Figure 2, it can be observed that Fe-MIL-88A has the largest crystal size, resulting in the highest number of Fe(III)CUS. Consequently, the activation performance of Fe-MIL-88A was superior to that of Fe-MIL-53 and Fe-MIL-101. Similarly, it can be concluded that the activation performance of Fe-MIL-101 is relatively poor.

To confirm the degradation mechanism of CIP, the active species under the four systems were identified. The EPR spectra of the four systems are shown in Figure 3B. The major free radicals, OH (symmetry center: g = 2.006, height ratio: 1:2:2:1), SO_4_^**−˙**^ (symmetry center: g = 2.006, height ratio: 1:1:1:1:1:1), were observed, indicating the presence of ·OH and SO_4_^**−˙**^in the reaction system (Chi et al., 2021b). Based on the peak intensity of the EPR spectra, the activation performance of the four Fe-based MOFs followed the order: Fe(II)-MOFs > Fe-MIL-88A > Fe-MIL-53>Fe-MIL-101, which was consistent with the research results on their degradation rate of CIP. Additionally, two other active species (O_2_^**−˙**^ and ^1^O_2_) may exist in advanced oxidation systems based on persulfate. Further EPR testing of the four systems, revealed that there was almost no presence of O_2_^**−˙**^ and ^1^O_2_ in the common Fe-based MOFs activated PDS system, which indicates that the influence of O_2_^**−˙**^ and ^1^O_2_ on CIP removal can be excluded. To further confirm the main active species in the reaction systems, a free radical quenching experiment was conducted based on the Fe(II)-MOFs activated PDS system, as shown in Figure 3C. It can be observed that the degradation rate of CIP significantly decreases when ethanol (EtOH) is added to the reaction system. Conversely, when t-butanol (TBA) is added, the degradation rate of CIP remains unchanged. This indicates that SO_4_^**−˙**^ is the main active species in the Fe-based MOFs activated PDS system, and ·OH only plays an auxiliary role.

In Fe-based MOFs activated PDS systems, the reusability of the catalyst is also an important factor for activation performance. The results are shown in Figure 3D. At the end of each cycle, the removal rates of CIP are 86.34%, 84.69%, 82.34%, 81.43%, and 80.12% respectively. The removal rate of CIP shows a downward trend with an increase in the cycle numbers, although the trend is not significant. This may be caused by several factors: a certain amount of catalyst may collapse and dissolve in the solution, which has an adverse impact on the removal rate of CIP (Fang et al., 2018). Therefore, it is crucial to summarize the factors affecting the stability of catalysts during Fe-based MOFs active PDS systems.

**FIGURE 4:**
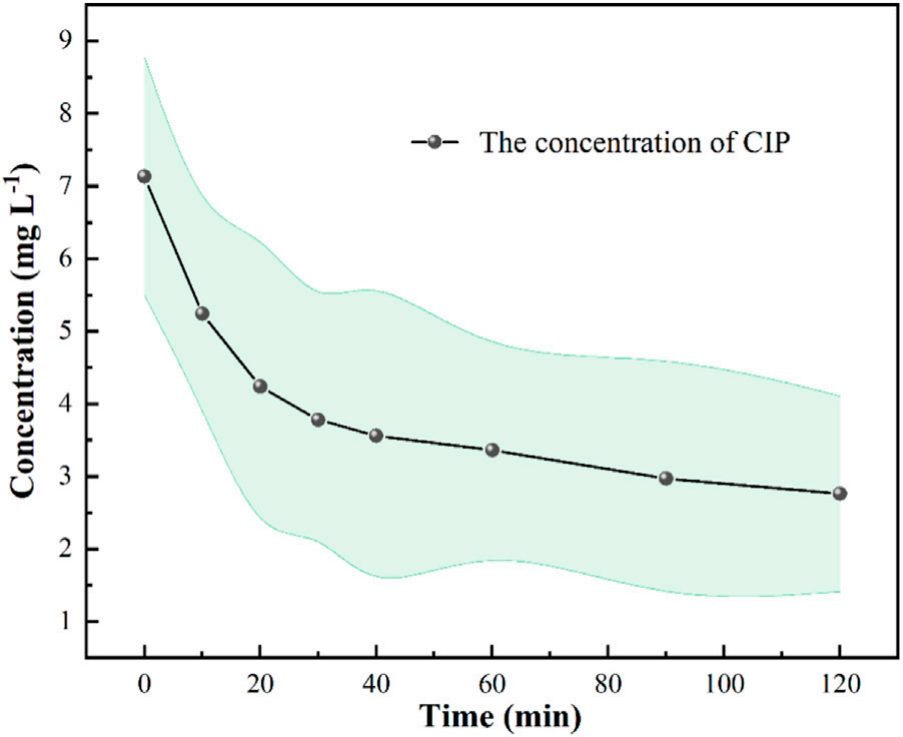
Fe based MOFs activate PDS to degrade CIP in actual wastewater. Experimental conditions: [PDS] = 0.036 mmol L^-1^, catalysts amount = 0.4 g L ^-1^, T = 25°C, ambient pH.

To evaluate the feasible application of this technology in real-world scenarios, we conducted degradation experiments using natural wastewater obtained from aquaculture farms (Figure 4). Our findings demonstrated that Fe-based MOFs can effectively activate PDS to degrade CIP in actual wastewater, indicating its significant potential in treating real wastewater. Moreover, we observed that the degradation effect of CIP in actual wastewater was inferior to that in experimental water preparation. This can be attributed to the presence of multiple ions and organic matter in actual wastewater, which negatively impacts the degradation process (Chi et al., 2020). Considering the activation performance of Fe-based MOFs, we can conclude that they exhibit substantial advantages in activating PDS to form sulfate radicals, highlighting their potential for industrial applications in activating advanced oxidation reagents for the removal of OMPs.

### Stability performance of Fe-based MOFs

**FIGURE 5:**
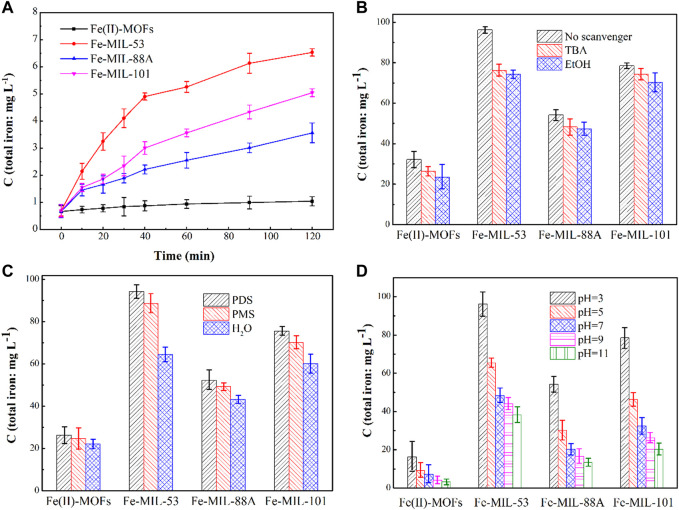
**(A)** The leachable Fe in the solutions in different iron-based MOFs/PDS systems. Experimental conditions: [CIP] = 0.036 mmol L^-1^, CIP: PDS = 1: 150, catalysts amount = 0.4 g L ^-1^, T = 25°C, ambient pH. **(B)** Influence of radical scavenger on CIP degradation in different Fe-based MOFs/PDS systems. Experimental conditions: catalysts amount = 1.0 g L ^-1^, [TBA]/[EtOH] = 0.4 mol L^-1^, T = 25°C, ambient pH. **(C)** Influence of oxidant on CIP degradation in different iron-based MOFs/PDS systems. Experimental conditions: catalysts amount = 1.0 g L ^-1^, T = 25°C, ambient pH. **(D)** Influence of pH on CIP degradation in different iron-based MOFs/PDS systems. Experimental conditions: [CIP] = 0.036 mmol L^-1^, CIP: PDS = 1: 150, catalysts amount = 1.0 g L ^-1^, T = 25°C.

In water environments, water molecules can attack the coordination bond between the metal central atoms and oxygen atoms in Fe-based MOFs, causing the crystal structure to collapse and some Fe atoms to dissolve (Burtch et al., 2014). Therefore, the stability of Fe-based MOFs can be indirectly assessed by measuring the concentration of Fe ions in the reaction solution. The amount of released Fe ions in four types of activated PDS systems based on Fe-based MOFs is shown in Figure 5A. All types of Fe-based MOFs exhibited a certain degree of Fe ion dissolution, indicating that they suffer damage during the activation process. Among them, the Fe(II)-MOFs system showed the least dissolution of Fe ions. This is because the presence of F ligands in Fe(II)-MOFs enhances the coordination bond between Fe and O atoms, thereby improving the stability performance of MOFs (Chi et al., 2019). Furthermore, although Fe-MIL-53, Fe-MIL-88A, and Fe-MIL-101 have the same elemental composition (Figure 2), there are differences in the dissolution of Fe ions. It is known that the presence of active species directly affects their activation performance. Hence, it can be speculated that the active species have a negative impact on stability performance. Comparing the results in Figure 3A, it can be observed that the concentration of dissolved Fe ions from Fe-based MOFs is roughly negatively correlated with their activation effect. In other words, higher reaction activity results in lower dissolution of Fe ions. Additionally, the dissolution of Fe ions in the Fe-MIL-101/PDS system is lower than that in the Fe-MIL-53/PDS system, but their relationship with reaction activation performance is exactly the opposite (Figure 3A). This suggests that the crystal structure of Fe-based MOFs has a certain influence on their stability performance. When the crystal structures are similar, a smaller crystal size indicates better stability performance (Fan et al., 2020).

To investigate the variations in the stability performance of Fe-based MOFs under different conditions, a series of control experiments were conducted. Quenching agents were added to the activated PDS systems based on Fe-based MOFs to examine the effect of active species on the dissolution of Fe ions. The duration of the experiment was 48 h, during which 0.0036 mmol CIP and 0.36 mmol PDS were added to the reaction system every 6 h. The concentrations of Fe ions in different reaction systems are shown in Figure 5B. As the quenching agents were introduced, the total concentration of Fe ions in the reaction system gradually decreased. Upon adding TBA, the concentrations of Fe ions in the Fe(II)-MOFs, Fe-MIL-88A, Fe-MIL-53, and Fe-MIL-101 systems decreased by 18.28%, 20.78%, 11.08%, and 5.34%, respectively. Similarly, with the addition of EtOH, the concentrations of Fe ions decreased by 21.25%, 22.86%, 12.87%, and 10.57%, respectively. The effective quenching of ·OH and SO_4_^**−˙**^ is attributed to EtOH, while TBA effectively quenches ·OH alone. Based on this, it can be inferred that the influence of ·OH is greater than that of SO_4_^**−˙**^ (Han et al., 2010). This is because SO_4_^**−˙**^ consists of electronegative groups, which are more likely to accumulate on the surface of CIP for oxidation reactions. Furthermore, it was observed that free radicals had the least impact on the concentration of dissolved Fe ions in the Fe(II)-MOFs activated PDS system. This suggests that the crystal structure of Fe(II)-MOFs exhibits better stability performance in advanced oxidation systems, owing to its more stable coordination structures.

The choice of oxidant reagent in advanced oxidation processes is an important factor that affects the activation performance of Fe-based MOFs. In this study, we selected similar persulfates, namely, Peroxodisulfate (PDS) and Peroxomonosulfate (PMS), for comparative testing. The duration of the experiment was 48 h, and three sets of experiments were conducted using reaction solutions of 100 mL H_2_O, 100 mL H_2_O+0.36 mmol PDS, and 100 mL LH_2_O+0.36 mmol PMS, respectively. Every 6 h, 0.36 mmol PDS/PMS was added to the reaction system. The total concentration of iron ions in each Fe-based MOFs system is shown in Figure 5C. When the reaction system consisted of H_2_O alone, the concentrations of dissolved Fe ions in the Fe(II)-MOFs, Fe-MIL-88A, Fe-MIL-53, and Fe-MIL-101 systems were 22.01 mg·L^−1^, 64.36  mg·L^−1^, 43.24  mg·L^−1^, and 60.11  mg·L^−1^, respectively. With the addition of the oxidant reagent (PDS/PMS), the concentration of dissolved Fe ions in the reaction system significantly increased. When PDS was added, the concentration of Fe ion dissolution increased by 18.12%, 46.39%, 20.79%, and 25.63%, respectively. Similarly, when PMS was added, the concentrations of Fe ion dissolution increased by 12.26%, 37.69%, 14.10%, and 16.63%, respectively. These results indicate that the oxidant reagent caused some damage to Fe-based MOFs, and the effect of PDS on Fe-based MOFs was greater than that of PMS. This can be attributed to the different oxidation properties of PDS and PMS. PDS molecules contain peroxide bonds (O-O), which confer strong oxidation ability, whereas PMS is an asymmetric oxide with high water solubility and mild oxidation ability (Cui et al., 2016). Therefore, PDS results in a stronger detrimental impact on the crystal structure of Fe-based MOFs.

In the advanced oxidation process based on PDS, the pH value plays a crucial role in influencing the production of active substances. Therefore, we hypothesized that the initial pH value would also have an impact on the dissolution of Fe ions. To test this hypothesis, we measured the concentration of dissolved Fe ions at different initial pH values (Figure 5D). The duration of the reaction was still 48 h, and every 6 h, 0.36 mmol of PMS was added to the reaction system. The influence of the pH value on the stability performance of the four types of Fe-based MOFs was found to be similar. As the pH value increased, the concentration of dissolved Fe ions in the reaction system significantly decreased. This indicates that the crystal structure of Fe-based MOFs was more susceptible to damage under acidic conditions. The higher likelihood of PDS undergoing activation reactions and generating active species under acidic conditions was responsible for this effect, leading to damage to the crystal structure of Fe-based MOFs (Figure 5B). Furthermore, it was observed that the pH value had the least effect on the concentration of dissolved Fe ions in the Fe(II)-MOFs system. This is because the crystal structure of Fe(II)-MOFs is relatively stable. The presence of F elements in the crystal structure enhances the interaction between the metal central atoms and organic ligands, thereby improving the stability performance of the MOFs crystal structure (Chi et al., 2019).

**FIGURE 6:**
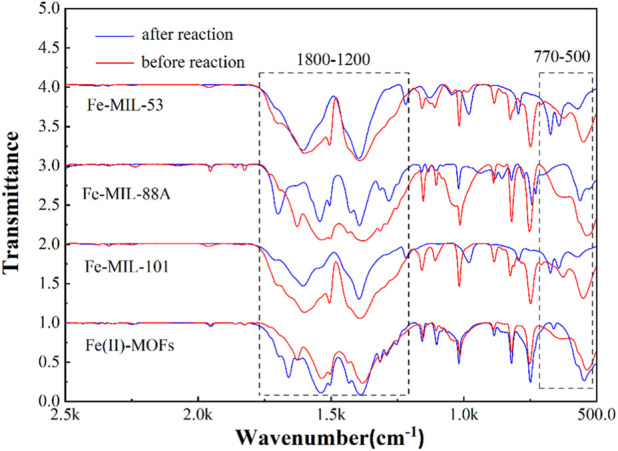
The FT-IR of as-prepared Fe-based MOFs and the reaction samples.

Based on the experimental results mentioned above, it was found that Fe(II)-MOFs exhibited the best stability performance in terms of crystal structure, while all types of Fe-based MOFs experienced damage during advanced oxidation processes. The crystal structure could be attacked by water molecules, active species, or H^+^. Therefore, the damage mechanism of the crystal structure was inferred by analyzing the surface functional groups of Fe-based MOFs before and after the activation reaction. The FT-IR spectra of Fe-based MOFs are shown in Figure 6. The intensity of the characteristic peak decreased to some extent after the reaction, indicating a general consumption effect on the functional groups in Fe-based MOFs during advanced oxidation processes (Zhang et al., 2020b). In detail, the most representative infrared characteristic peak regions were observed between 1,200 and 1,800 cm^−1^ and 500–700 cm^−1^. The C-H bending vibration on the benzene ring was represented at approximately 1,501 and 1,380 cm^−1^, and the weakened intensity indicated some disintegration of the benzene ring structure of MOFs. However, the characteristic peaks at approximately 1,501 and 1,380 cm^−1^ still remained clear before and after the reaction, demonstrating that the crystal structure of Fe-based MOFs remained intact while explaining the damage to the benzene ring (Chen et al., 2019). The characteristic peak at approximately 500–770 cm^−1^ represented the tensile vibration mode of Fe-O (Liu et al., 2019). The changes in this part of the characteristic peak for different Fe-based MOFs were complex, including the disappearance or transformation of characteristic peaks, indicating complex changes in the coordination bond of Fe in MOFs. The attack of water molecules and free radicals focused on the coordination bond of Fe, weakening the bond energy between the Fe atom and O atom in Fe-based MOFs, leading to the collapse of the crystal structure. Additionally, the characteristic peaks at approximately 1,601 cm^−1^ represented the *δ* and *ω* C-H stretching modes and the C=C stretching mode influenced by F (Özdemir Tarı et al., 2018). In the FT-IR spectra of Fe(II)-MOFs, the characteristic peak at approximately 1,601 cm^−1^ was consistently present, and the characteristic peak of Fe(II)-MOFs before and after the reaction showed minimal change. This result indicated that the presence of F ligands enhanced the bond energy between Fe atoms and O atoms, thereby improving the stability performance of Fe-based MOFs. Based on the analysis of the stability performance of Fe-based MOFs, we can summarize two methods to enhance their stability performance, which are crucial for their application in removing OMPs: 1) introducing ligands to enhance the bond energy between Fe atoms and O atoms, and 2) modifying the hydrophobicity of Fe-based MOFs to reduce the damage caused by water molecules to the crystal structure.

## Conclusion

In this study, four types of Fe-based MOFs with different structures were synthesized, namely, Fe(II)-MOFs, Fe-MIL-53, Fe-MIL-88A, and Fe-MIL-101. The crystal structure and surface morphology analysis confirmed that all types of Fe-based MOFs had complete crystal structures. Moreover, all types of Fe-based MOFs were capable of effectively activating PDS to degrade CIP in wastewater. The results revealed that Fe(II)-MOFs exhibited the highest degradation rate constant (0.155 min^−1^) due to the presence of Fe(II) CUS as the main active site. However, the process of Fe-based MOFs activating PDS for CIP degradation had a negative impact on the stability of the MOFs’ structure. The activated species, oxidant reagents, and pH levels all caused certain damage to the crystal structure of Fe-based MOFs. The findings demonstrated the following: 1) ·OH had a stronger impact on the crystal structure than SO_4_^**−˙**^; 2) PDS had a greater influence compared to PMS; and 3) the crystal structure suffered severe damage under acidic conditions. Furthermore, water molecules, free radicals, and H^+^ attacked the coordination bond between the metal atom center and the organic ligand, resulting in the dissolution of Fe ions.

## Data Availability

The original contributions presented in the study are included in the article/supplementary material, further inquiries can be directed to the corresponding author.
